# Supplementary material for: Genome-Wide Characterization of DGATs and Their Expression Diversity Analysis in Response to Abiotic Stresses in Brassica napus
Source: Plants (Basel). 2022 Apr 25;11(9):1156. doi: 10.3390/plants11091156 (PMC9104862; doi:10.3390/plants11091156)
Supplement: Supplementary file 1 [file plants-11-01156-s001.zip › Supplementary Figure legends.pdf]

Figure S1. Schematic representation of *DGAT* gene distribution on *Brassica napus, Brassica rapa,* and *Brassica oleracea* chromosomes. (A) The distribution of *BnaDGATs* on *B. napus* chromosomes. (B) The distribution of *BraDGATs* on *B. rapa* chromosomes. (C) The distribution of *BolDGATs* on *B. oleracea* chromosomes. The detailed chromosome locations of *DGATs* in *B. napus*, *B. rapa*, and *B. oleracea* were acquired from the GFF genome files downloaded from the *B. napus* genomic database (BnPIR, http://cbi.hzau.edu.cn/bnapus (accessed on 02 March 2021)) and *Brassica* Database (BRAD, http://brassicadb.cn/#/ (accessed on 11 October 2021)), respectively, and the predicted locations were mapped on the chromosomes by using TBtools software. The gene names of *DGAT1s, DGAT2s* and *DGAT3s* are indicated with red, green and blue, respectively.

Figure S2. Gene structures of DGAT family members in plants. **(A**) Phylogenetic tree. DGAT protein sequences were used to construct the phylogenetic tree by MEGA11 software with the neighbour-joining method and 2000 bootstrap replications. (**B**) Gene structures. The gene structures were constructed using the Gene Structure Display Serve tool (http://gsds.gao-lab.org/ (accessed on 20 October 2021)) [53]. BnaDGAT1, BnaDGAT2 and BnaDGAT3 were cloned from *B. napus* ZS11. *DGATs* of other species were selected from the corresponding genome databases (Table S1). Black boxes denote exons within coding regions, and the lines connecting them represent introns. Grey boxes indicate the upstream or downstream regions of the CDS of DGAT genes. The length of boxes and lines represent the size of the corresponding exon and intron, respectively.

Figure S3. The conserved motifs of DGAT family members in plants
(**A**) Phylogenetic tree. DGAT protein sequences were used to construct the phylogenetic tree by MEGA11 software with the neighbour-joining method and 2000 bootstrap replications. (**B** and **C**) The top 20 conserved motifs distributed in DGATs. Amino acid sequences of DGATs were submitted to the MEME program (Version 5.4.1, https://meme-suite.org/meme/tools/meme (accessed on 21 October 2021)) with the maximum motif search set to 20 and other parameters set to default to identify the conserved protein motifs [105]. Then, the conserved motifs were visualized by using TBtools software [103]. *BnaDGAT1s*, *BnaDGAT2s* and *BnaDGAT3s* were cloned from *B. napus* ZS11. The protein, genomic DNA and CDSs of DGAT family members in *B. oleracea*, *B. rapa*, *B. nigra*, *B. juncea*, *B. carinata, A. thaliana*, *A. hypogaea*, *G. max*, *R. communis*, *O. sativa*, *Z. mays*, *S. italica*, *M. truncatula*, *B. distachyon* and *J. curcas* were blasted and selected from their corresponding genome databases (Table S2).
