# Supplementary figures and images for: Genome-Wide Characterization of DGATs and Their Expression Diversity Analysis in Response to Abiotic Stresses in Brassica napus
Source: Plants (Basel). 2022 Apr 25;11(9):1156. doi: 10.3390/plants11091156 (PMC9104862; doi:10.3390/plants11091156)

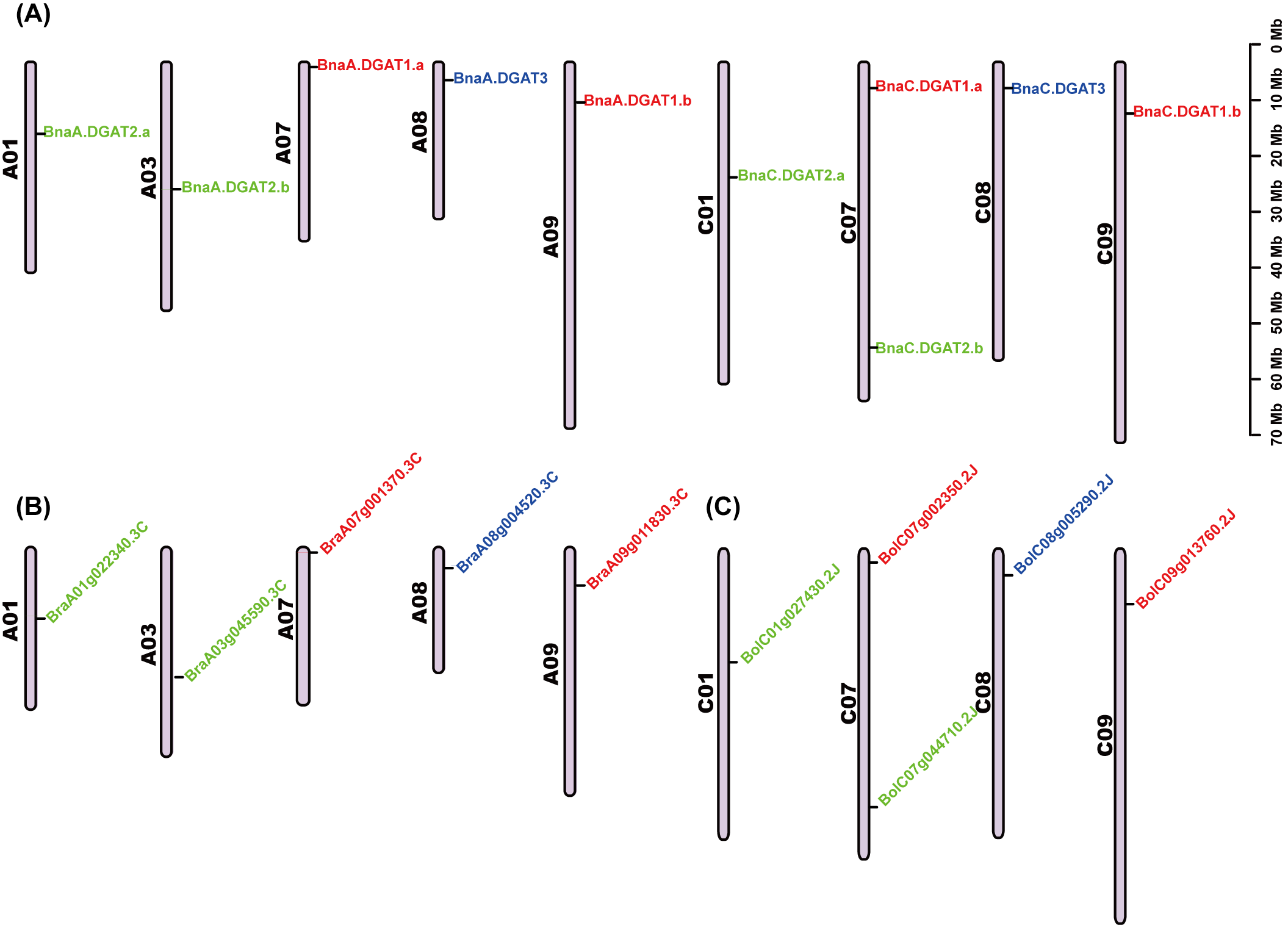

Supplement: Supplementary file 1 [file plants-11-01156-s001.zip › Figure S1.tif]

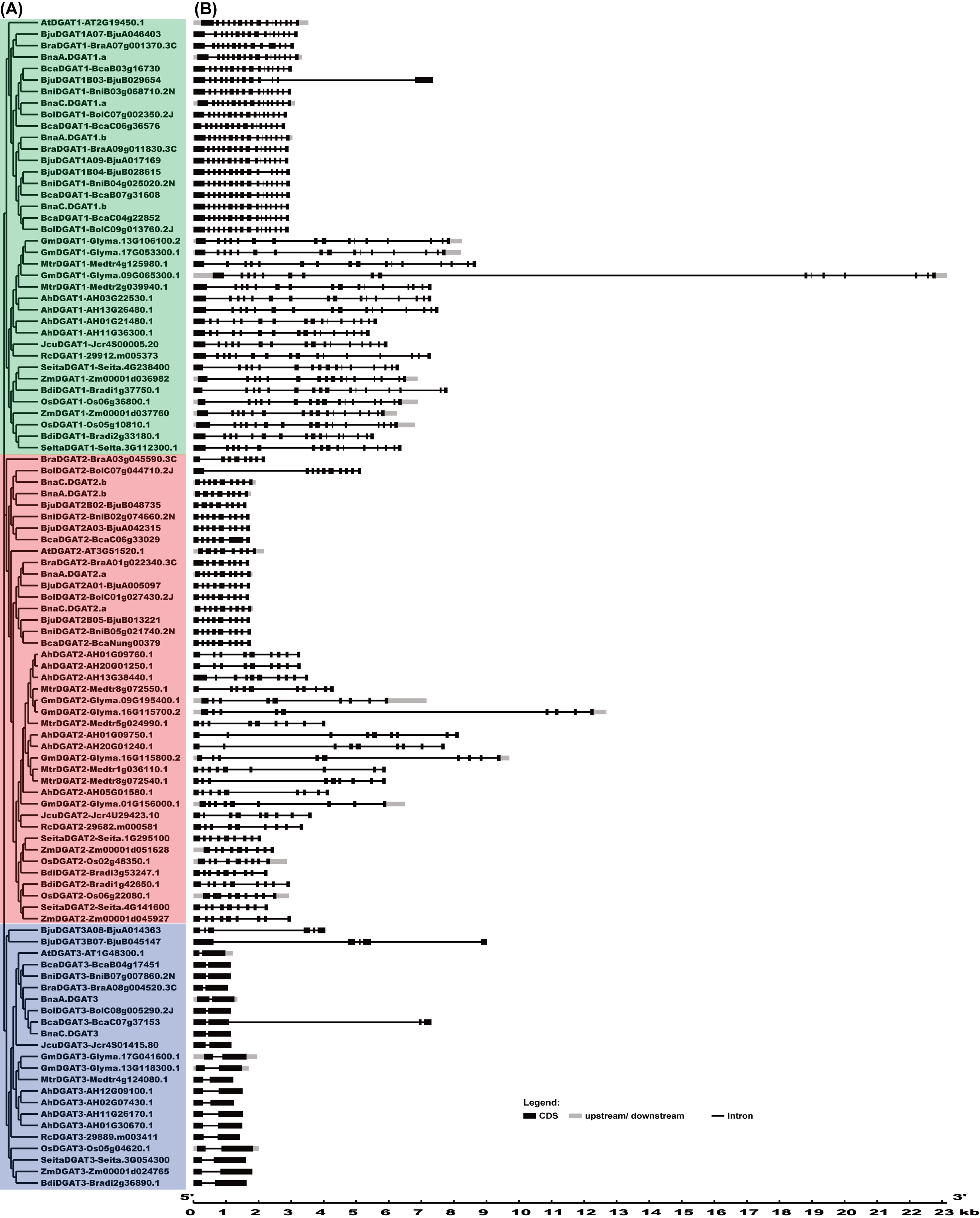

Supplement: Supplementary file 1 [file plants-11-01156-s001.zip › Figure S2.tif]

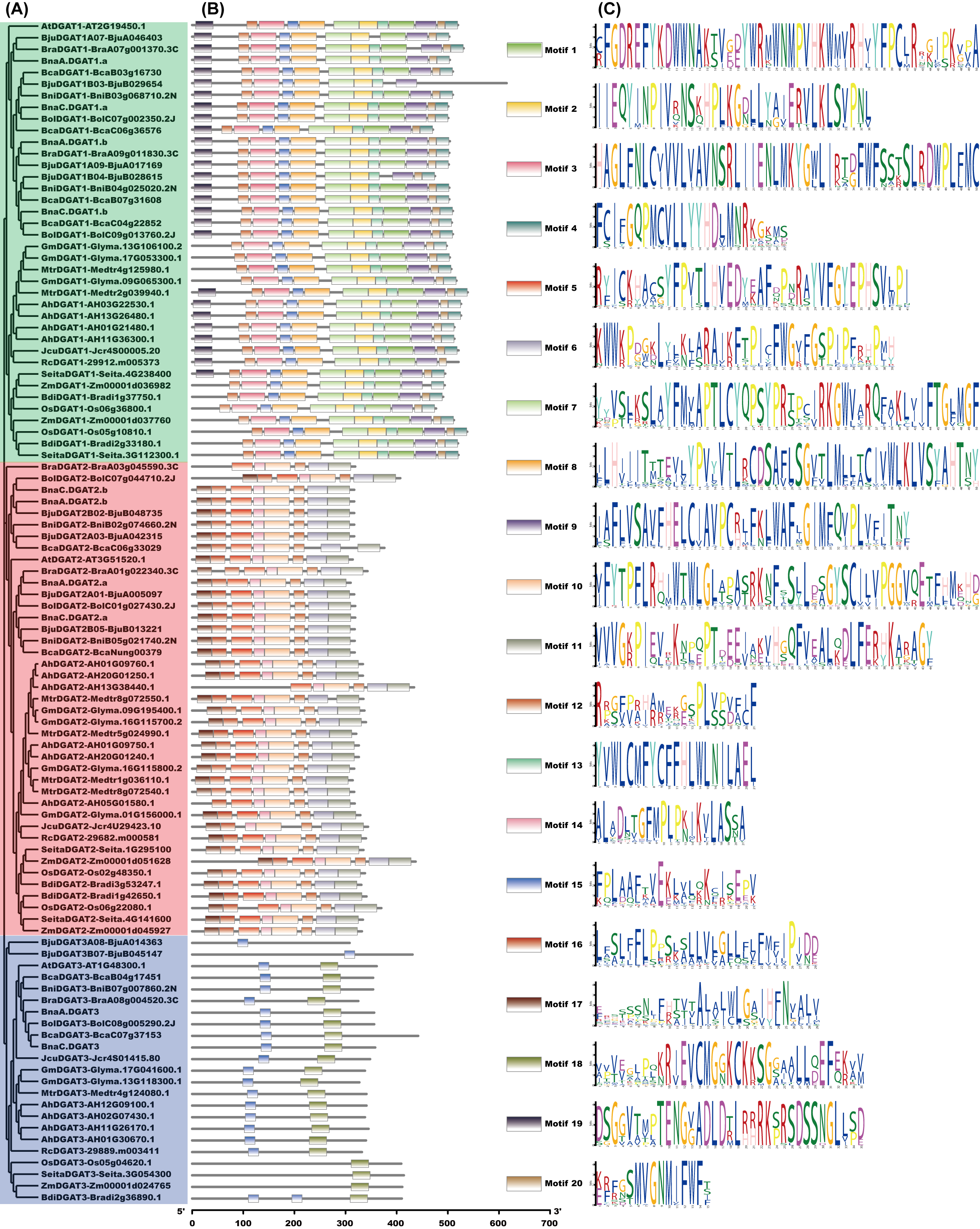

Supplement: Supplementary file 1 [file plants-11-01156-s001.zip › Figure S3.tif]
